# Role of Ca^2+^ in the rapid cooling-induced Ca^2+^ release from sarcoplasmic reticulum in ferret cardiac muscles

**DOI:** 10.1007/s12576-012-0203-1

**Published:** 2012-03-20

**Authors:** Etsuko Tanaka, Masato Konishi, Satoshi Kurihara

**Affiliations:** 1 Department of Cell Physiology, The Jikei University School of Medicine, 3-25-8 Nishi-shinbashi, Minato-ku, Tokyo, 105-8461 Japan; 2 Department of Physiology, Tokyo Medical University, 6-1-1 Shinjuku, Shinjuku-ku, Tokyo, 160-8402 Japan

**Keywords:** Calcium, Sarcoplasmic reticulum, Rapid cooling, Ca^2+^-induced Ca^2+^ release

## Abstract

Rapid lowering of the solution temperature (rapid cooling, RC) from 24 to 3°C within 3 s releases considerable amounts of Ca^2+^ from the sarcoplasmic reticulum (SR) in mammalian cardiac muscles. In this study, we investigated the intracellular mechanism of RC-induced Ca^2+^ release, especially the role of Ca^2+^, in ferret ventricular muscle. Saponin-treated skinned trabeculae were placed in a glass capillary, and the amount of Ca^2+^ released from the SR by RC and caffeine (50 mM) was measured with fluo-3. It was estimated that in the presence of ATP about 45% of the Ca^2+^ content in the SR was released by RC. The amount of SR Ca^2+^ released by RC was unchanged by the replacement of ATP by AMP-PCP (a non-hydrolysable ATP analogue and agonist for the ryanodine receptor but not for the Ca^2+^ pump of SR), suggesting that the suppression of the Ca^2+^ pump of SR at low temperature might not be a major mechanism in RC-induced Ca^2+^ release. The free Ca^2+^ concentration of the solution used for triggering RC-induced Ca^2+^ release was estimated to be only about 20 nM with fluo-3 or aequorin. When this solution was applied to the preparation at 3°C, only a small amount of Ca^2+^ was released from SR presumably by the Ca^2+^-induced Ca^2+^ release (CICR) mechanism. Thus, in mammalian cardiac muscles, RC releases a part of the (<50%) stored Ca^2+^ contained in the SR, and the mechanism of RC-induced Ca^2+^ release may differ from that of CICR, which is thought to play a role in frog skeletal muscle fibres that express ryanodine receptors of different types.

## Introduction

Rapid lowering the solution temperature is known to induce contracture in skeletal, cardiac and smooth muscles (rapid cooling contracture, RCC) [1–3]. Among these muscles, treatment of the preparation with low concentrations of caffeine is required for RCC in frog skeletal muscle [4]. In RCC of frog skeletal muscle, caffeine releases a small amount of Ca^2+^ from the sarcoplasmic reticulum (SR) by the Ca^2+^-induced Ca^2+^ release (CICR) mechanism, and low temperature inhibits Ca^2+^ uptake by SR. Thus, it was supposed that both caffeine and low temperature synergistically increase Ca^2+^ concentration around the SR and work to facilitate the CICR mechanism, leading to a massive Ca^2+^ release from SR [5].

However, in mammalian cardiac muscles, pre-treatment with caffeine is not required for RCC [3], and the intracellular Ca^2+^ concentrations before rapid cooling (RC) measured in intact ferret papillary muscles do not correlate with the amount of Ca^2+^ released by RC [6], suggesting that the mechanism of RC-induced Ca^2+^ release differs from that of CICR. Thus, the Ca^2+^ release mechanism by RC in cardiac muscle may differ from that in skeletal muscle.

In this study, we investigated the intracellular mechanism of RC-induced Ca^2+^ release in ferret ventricular muscle. We focused on whether the RC-induced Ca^2+^ release in cardiac muscle could be explained by CICR. For this purpose, quantitative measurement of Ca^2+^ concentrations around SR before RC is essential. Therefore, we used the previously established methodology with saponin-treated skinned preparations in which the SR functions are known to be well preserved [7, 8]. The saponin-skinned trabeculae were placed in a glass capillary, and various solutions with different compositions were directly applied to the SR to estimate the amount of released Ca^2+^ from the SR and Ca^2+^ content in SR.

A part of the results was presented in abstract form [9].

## Materials and methods

All experiments were performed in accordance with the Guidelines on Animal Experimentation of The Jikei University School of Medicine.

### Preparations

Male ferrets weighing 600–1,200 g were anaesthetised by intraperitoneal injection of pentobarbitone (150 mg kg^−1^), and the hearts were quickly removed. The blood in the heart was washed out by retrograde perfusion of normal Tyrode’s solution at 30°C through the aorta. Then, the right ventricle wall was quickly opened, and papillary muscles or trabeculae were dissected out in normal Tyrode’s solution at 30°C. The papillary muscles or trabeculae were cut along the longitudinal axis in the relaxing solution with 10 mM EGTA and 4.6 mM ATP at 4°C. The diameter of the preparation was 180–305 μm (241 ± 5 μm, mean ± standard error of the mean, *n* = 38) and the length was 2.1–6.0 mm (3.8 ± 0.2 mm, *n* = 38). The preparation was tied at both ends to a tungsten wire (diameter 50 μm, length 12 mm) with silk thread and treated with saponin (10 μg/ml) in the relaxing solution for 30–40 min. The saponin-treated preparation was then inserted into a glass capillary tube (internal diameter 500–600 μm) and fixed inside the capillary by bending the ends of the tungsten wire. The fluorescence signal was measured in the central portion (1 mm long) of the preparation.

### Experimental apparatus

**Fig. 1:**
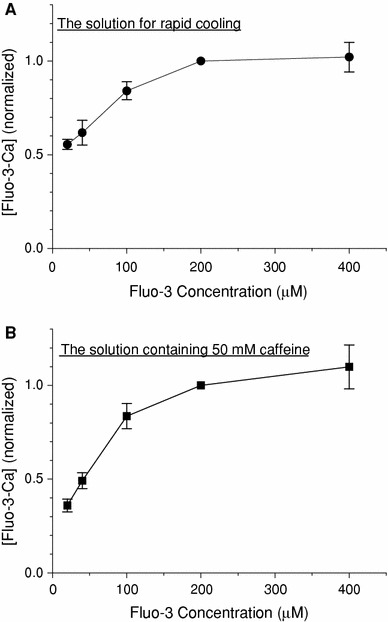
The relation between fluo-3 concentration and [Ca · Fluo-3] in the presence or absence of 50 mM caffeine. After Ca^2+^ loading, Ca^2+^ was released from the SR into a glass capillary lumen by rapid cooling (**a**) or 50 mM caffeine (**b**). G0M1.5F and G0RM0CafF solutions (see Table 1) were used for **a** and **b**, respectively, at 3°C. The fluorescence signal was measured with five different concentrations of fluo-3 and was calibrated in terms of Δ[Fluo-3-Ca] (see Eq. 1 in text). *Each symbol* represents mean ± SEM (*n* = 5)

The experimental apparatus used was slightly modified from the original apparatus shown in Fig. 1 of Kawai and Konishi [7]. The glass capillary containing the preparation was placed on an inverted microscope (IMT2-F4, Olympus, Tokyo, Japan). One end of the glass capillary was connected to a silicon tube as a solution inlet. The tube was passed through either one of two heat exchangers set at 24 and 3°C, and was connected to a step-motor-controlled multiposition (16 channels) valve (EMT-O-CSD16UWP-HC, Valco Instruments Co. Inc., Houston, TX, USA) for the selection of perfusing solution. A multiposition valve actuator for the rotation of ports was carried out using a personal computer (PC-9801NS/A, NEC, Tokyo, Japan). The other end of the capillary (a solution outlet) was linked to a peristaltic pump (Perista Bio-minipump, ATTO Co., Tokyo, Japan) via a silicon tube. The rate of solution flow was controlled by changing the rotor speed of the pump.

Excitation light from a xenon lamp (UXL-75XB, Ushio Inc., Tokyo, Japan) was passed through a 480-nm filter (excitation filter, half bandwidth 15 nm, RDF480, Omega Optical Inc., Brattelboro, VT, USA), and the fluorescence emitted from the capillary of a wavelength longer than 530 nm was detected through a barrier filter (emission filter, OG530, Melles Griot, Carlsbad, CA, USA) with a photomultiplier (R03-RB12M, Hamamatsu Photonics). The output of the photomultiplier was fed to a photometer system (P101/P102, Nikon Co., Tokyo, Japan). Storage and analysis of the data were carried out on a personal computer (PC-9801DX, NEC, Japan). The A/D converter, pump, pinch valve and shutter for the excitation light (EC-601, Copal, Tokyo, Japan) were controlled by the stimulator pulses of a preset sequence (DPS-1300D, Dia Medical System Co., Ltd., Tokyo, Japan).

### Solutions and chemicals

The normal Tyrode’s solution used for dissection was as follows (mM): Na^+^, 135; K^+^, 5; Ca^2+^, 2; Mg^2+^, 1; Cl^−^, 102; HCO_3_^−^, 20; HPO_4_^2−^, 1; acetate, 20; glucose, 10; insulin, 5 units l^−1^; pH, 7.3–7.4 at 30°C when equilibrated with 5% CO_2_ + 95% O_2_.

The composition of the relaxing solution used for cutting out the preparations and for treating the preparations with saponin was as follows (mM): K_2_ATP (adenosine 5′-triphosphate, dipotassium salt), 5.2; EGTA (ethylene glycol-bis (2-aminoethyl)-N,N,N′,N′-tetraacetic acid), 10.0; PIPES (piperazine-N-N′-bis (2-ethane sulfonic acid)), 20.0; Mg^2+^, 1.5; MgMs_2_, 5.6; KMs, 90.8; NaMs, 10.0; Leupeptin, 10 μg/ml; pH, 7.0 adjusted by KOH at 4°C.

The composition of the solutions used for measuring fluorescence is shown in Table 1. Ionic constituents were computed by solving multi-equilibrium equations using binding constants compiled by Martell and Smith [10]. All solutions contained 20 mM PIPES, 5 μM CCCP (carbonyl cyanide *m*-chlorophenylhydrazone) and 10 μM DCB (2′,4′-dichlorobenzamil hydrochloride). Ionic strength was 0.2 M. Loading and assay solutions contained 10–20 μg/ml Leupeptin. pH was adjusted to 7.0 by KOH at 3 or 24°C. K_2_ATP, AMP (adenosine 5′-monophosphate), K_2_AMP-PCP (β,γ-methyleneadenosine-5′-triphosphate), saponin and CCCP were obtained from Sigma Chemical Co. (St. Louis, MO, USA). DCB and fluo-3 (pentapotassium salt) were purchased from Invitrogen (OR, USA). PIPES and caffeine were obtained from Nacalai Tesque, Inc. (Kyoto, Japan), EGTA from Fluka Chemie AG of Sigma-Aldrich Co. and Leupeptin from Peptide Institute, Inc. (Osaka, Japan). All chemicals used were of analytical grade. Aequorin was purchased from Dr. J.R. Blinks.

**Table 1:** Composition of solutions

Solution	K_2_ATP (mM)	AMP (mM)	EGTA (mM)	CaMs_2_ (mM)	Mg^2+^ (mM)	MgMs_2_ (mM)	KMs (mM)	NaMs (mM)	Caffeine (mM)	Fluo-3 (μM)
Load: 24°C										
CaG1M1.5	4.1	0	1	0.4	1.5	5.5	117.5	10	0	0
Wash: 24°C										
G1R	0	0	1	0	1.5	1.6	143.9	10	0	0
G10R	0	0	10	0	1.5	2.1	116.5	10	0	0
Pre-assay: 24°C										
G0RM1.5F	0	0	0	0	1.5	1.5	147	10	0	20
G0RM0F	0	0	0	0	0	0	151.5	10	0	20
Assay: 3°C										
G0M1.5F	4.6	0	0	0	1.5	5.5	120.7	10	0	20
G0RM0CafF	0	25	0	0	0	0	95.1	10	50	20

All solutions contained 10 mM PIPES, 5 μM CCCP and 10 μM DCB. Ionic strength was 0.2 M. Load and assay solutions contained 10–20 μg/ml leupeptin. pH was adjusted to 7.0 by KOH at 3 or 24°C. The meanings of the abbreviations are as follows: G0, 1, 10; 0, 1, 10 mM EGTA, M0, 1.5; 0, 1.5 mM free Mg^2+^, R; no ATP, Ca; pCa 6.5, Caf; 50 mM caffeine, F; 20 μM fluo-3

### Estimation of total Ca^2+^ released from the SR

After RC, Ca^2+^ was released from the SR to the capillary lumen filled with the “assay” solution containing 20 μM fluo-3 (G0M1.5F, Table 1). The fluorescence intensity of fluo-3 in the assay solution was low in the absence of Ca^2+^ release from the SR (*F*_0_), but it rose to higher levels (*F*) after the onset of Ca^2+^ release from the SR. To quantify the amount of released Ca^2+^, we used the method previously established in our laboratory [7]. At the end of each experiment, the assay solution plus 1 mM Ca^2+^ was introduced into the capillary that contained the muscle preparation to estimate the fluorescence of Ca^2+^-bound fluo-3 (*F*_max_). The fluorescence of Ca^2+^-free fluo-3 (*F*_min_) was measured by introducing the assay solution plus 1 mM EGTA. *F*_min_ was slightly lower than *F*_0_ because of slight contamination of Ca^2+^ in the assay solution (1.3 μM estimated with fluo-3). The change in fluo-3 fluorescence after Ca^2+^ release (*F* − *F*_0_), could be calibrated in terms of the change in concentration of Ca^2+^-bound fluo-3 (Δ[Fluo-3 · Ca]) by the following equation:

1 $$ \Updelta [{\text{Fluo - 3}} \cdot {\text{Ca}}] = [{\text{Fluo - 3}}]_{\text{tot}} (F - F_{0} )/(F_{\max } - F_{\min } ), $$

where [Fluo-3]_tot_ was the total fluo-3 concentration in the assay solution and was 20 μM in the present study.

The amount of Ca^2+^ released from the SR, defined as the change in total Ca^2+^ concentration in the capillary lumen (Δ[Ca]_tot_), could be expressed as the sum of changes in concentrations of free Ca^2+^ (Δ[Ca^2+^]), Ca^2+^ bound to fluo-3 (Δ[Fluo-3 · Ca]) and Ca^2+^ bound to other buffers (e.g., intrinsic intracellular buffers and ATP in the solution) (Δ[B · Ca]):

2 $$ \Updelta [{\text{Ca}}]_{\text{tot}} = \Updelta [{\text{Ca}}^{{ 2 { + }}} ] + \Updelta [{\text{Fluo - 3}} \cdot {\text{Ca}}] + \Updelta [{\text{B}} \cdot {\text{Ca}}]. $$

When the fluo-3 concentration is high enough to predominantly bind released Ca^2+^ (i.e., Δ[Fluo-3 · Ca] ≫ Δ[Ca^2+^] + Δ[B · Ca]), Δ[Ca]_tot_ could be approximated with Δ[Fluo-3 · Ca]. Figure 1a shows the relation between the fluo-3 concentration in the assay solution (G0M1.5F, Table 1) and Δ[Fluo-3 · Ca]. After the SR was fully loaded with Ca^2+^, Ca^2+^ release was induced by RC. The calibrated Δ[Fluo-3 · Ca] increased as the fluo-3 concentration was increased up to 200 μM, but was saturated at higher fluo-3 concentrations (200–400 μM). The result suggested that the fluo-3 concentration of 200 μM was high enough to predominantly bind released Ca^2+^ from the SR and that Δ[Fluo-3 · Ca] obtained at high fluo-3 concentrations (≥200 μM) could be an index of the amount of Ca^2+^ released from the SR (i.e., Δ[Ca]_tot_ expressed in units of μM in the capillary space). With 20 μM fluo-3 primarily used in the present study for economic reasons, on the other hand, Δ[Fluo-3 · Ca] had to be scaled to attain the value that would be obtained if the fluo-3 concentration was sufficiently high (≥200 μM). The scaling factor of 1.80 estimated from Fig. 1a was used in the following sections.

We used the other assay solution that contained 50 mM caffeine and 25 mM AMP (G0RM0CafF, Table 1) to release all Ca^2+^ in the SR. High concentrations of caffeine and AMP should maximise Ca^2+^ release, and the Ca^2+^ pump was expected to be inactive in the absence of ATP. Because caffeine significantly influences fluo-3 fluorescence and affinities of intracellular buffer sites for Ca^2+^, we carried out a separate set of calibrations of fluo-3 fluorescence signals; at the end of each experiment, *F*_max_ and *F*_min_ were estimated by the introduction of the G0RM0CafF assay solution plus 1 mM Ca^2+^ and the assay solution plus 1 mM EGTA, respectively. The relation between the fluo-3 concentration and Δ[Fluo-3 · Ca] was constructed in the G0RM0CafF assay solution (Fig. 1b). Then Δ[Fluo-3 · Ca] was calculated by Eq. 1 and was scaled by a factor of 2.78 (estimated from Fig. 1b) to yield Δ[Ca]_tot_.

### Measurement of free Ca^2+^ concentration in the solutions for rapid cooling

The free Ca^2+^ concentration of the solution for RC (G0M1.5F, Table 1) was measured with two different methods. The first method used fluo-3 fluorescence measured in the glass capillary that contained the muscle preparation without loading SR with Ca^2+^, as described below in the next section (protocol D1, Fig. 3). We calculated [Ca^2+^] with

3 $$ [{\text{Ca}}^{2 + } ] = K_{\text{d}} \, [(F_{0} - F_{\min } )/(F_{\max } - F_{0} )], $$

where *K*_d_ denotes a dissociation constant of fluo-3 for Ca^2+^. For the value of *K*_d_, we used 0.476 μM determined previously at 22°C [7]. Note that little temperature dependence of the *K*_d_ value has been reported for Ca^2+^-fluo-3 bindings [11].

The second method used a Ca^2+^ photoprotein, aequorin; aequorin luminescence of the G0M1.5F solution before application to the preparation was measured in vitro at 3°C in the absence of the preparation. Aequorin was dissolved in the solution containing 150 mM KCl and 10 mM PIPES at pH 7.0 to a final concentration of 150 μM. The luminescence was converted to [Ca^2+^] using the following equation [12];

4 $$ [{\text{Ca}}^{2 + } ] = (L/L_{ \max } )^{1/n} + (L/L_{\max } )^{1/n} \times (K_{\text{TR}} - 1)/\left( {K_{\text{R}} (1 - (L/L_{\max } )^{1/n} )} \right) $$

where *L* is the aequorin light obtained from the solutions. *L*_max_ is the maximal light at saturating Ca^2+^. For constants *n*, *K*_R_ and *K*_TR_, we used, respectively, 3.11, 3.62 × 10^6^ and 85.62 obtained previously at 3°C (see Methods in [6]).

### Experimental protocol

SR was loaded with Ca^2+^ by incubation of the preparation at pCa 6.5 in the loading solution (CaG1M1.5, Table 1) for 2 min at 24°C, the condition optimised to achieve nearly full loading of the SR as in preliminary experiments (data not shown).

1. Measurements of the fractional Ca^2+^ release by RC

**Fig. 2:**
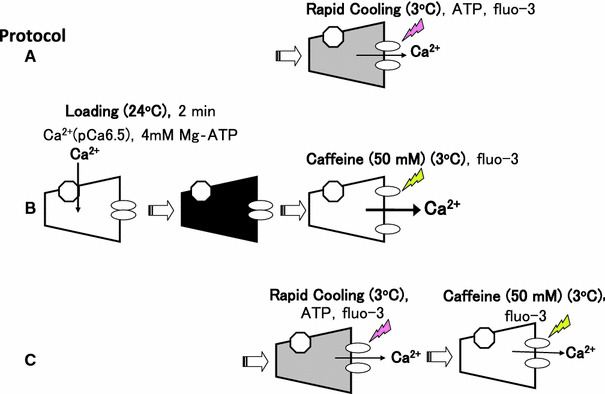
Overview of protocols. After loading SR with Ca^2+^ at pCa 6.5 in the solution containing 4.1 mM ATP (CaG1M1.5, see Table 1) for 2 min at 24°C, Ca^2+^ release was triggered by rapid cooling (protocol **A**), 50 mM caffeine (protocol **B**), and rapid cooling followed by 50 mM caffeine (protocol **C**). Ca^2+^ released from the SR was bound to fluo-3 in a glass capillary lumen, and the resultant fluorescence signal was measured as described in the text. Protocol **A** Temperature of a solution was lowered from 24 to 3°C within 3 s with simultaneous application of 4.6 mM ATP and 20 μM fluo-3 (G0M1.5F, see Table 1). Protocol **B** A solution that contained 50 mM caffeine, 25 mM AMP and 20 μM fluo-3 (G0RM0CafF, see Table 1) was applied at 3°C. Protocol **C** Rapid cooling (temperature of G0M1.5F lowered to 3°C) and 50 mM caffeine (G0RM0CafF) were sequentially applied. The meanings of *dark* and *shaded* trapezoids are full Ca^2+^ and decreased Ca^2+^ in SR, respectively

**Fig. 3:**
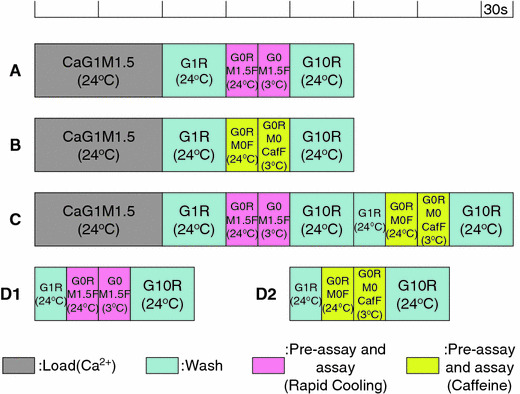
Timetable for solution exchange. Protocol **A**–**C** Correspond to, respectively, protocol **A**–**C** in Fig. 2. Protocol **D1** and **D2**, which lack the Ca^2+^ loading step, provide the “background” for the RC and caffeine assays, respectively

*Protocol A* After loading SR with Ca^2+^ and washout of the Ca^2+^ in the preparation with the Ca^2+^-free solution (G1R) at 24°C for 1 min, fluo-3 was introduced (G0RM1.5F), and the bathing solution temperature was lowered from 24 to 3°C within 3 s (G0M1.5F, Table 1). Ca^2+^ was released into a glass capillary (RC-induced Ca^2+^ release), and the fluorescent Ca^2+^ signal was measured (A in Figs. 2, 3).

*Protocol B* After Ca^2+^-loading and washing the preparation in the same manner as in protocol A, fluo-3 was introduced (G0RM0F, Table 1), and the total Ca^2+^ content in SR was released by 50 mM caffeine at 3°C (B in Figs. 2, 3).

*Protocol C* When 50 mM caffeine was applied to the preparation following RC-induced Ca^2+^ release, residual Ca^2+^ in SR was measured (C in Figs. 2, 3). In protocol C, the preparation was washed for 1.5 min (by G10R for 1 min and by G1R for 30 s) between RC and the application of 50 mM caffeine.

Protocols D1 and D2 lack SR Ca^2+^ loading steps, but otherwise were very similar to protocols A and B, respectively (Fig. 3).

For estimation of the amount of Ca^2+^ released by RC or caffeine, the fluo-3 fluorescence signals obtained without SR Ca^2+^ loading (protocol D1 for RC, and protocol D2 for caffeine) were subtracted from the fluorescence signals obtained with SR Ca^2+^ loading (protocol A or C for RC; protocol B or C for caffeine). We counted an average of 5 s around the peak of the subtracted signal.

The fractional SR Ca^2+^ release by RC was calculated using the values obtained from protocol A and B.

2. Effect of adenine nucleotides on Ca^2+^ release by RC

Protocol A was modified to study effects of ATP on RC-induced Ca^2+^ release. For removal of ATP, the assay solution of protocol A (G0M1.5, Table 1) was replaced by G0RM1.5 that did not contain ATP. When AMP-PCP, a non-hydrolysable ATP analogue, was used, 4.6 mM K_2_ATP in the assay solution (G0M1.5F) was simply substituted with 4.6 mM K_2_AMP-PCP.

3. Measurement of the amount of Ca^2+^ released by the CICR mechanism at 3°C

The protocol was identical to protocol A (above), except for modifications of the solution temperatures. Following Ca^2+^ loading in SR at 24°C for 2 min, the preparation in a capillary was washed with the Ca^2+^-free solution (G1R, Table 1), which contains neither ATP nor Ca^2+^, for 1 min. During this period, the temperature of the solution was slowly lowered from 24 to 3°C. After introduction of fluo-3 at 3°C (G0RM1.5F, Table 1), the solution for RC containing ATP and fluo-3 (G0M1.5F, Table 1) was applied at 3°C to the pre-cooled preparation. The change of fluo-3 fluorescence in a glass capillary was measured as described above for protocol A.

### Statistical analysis

We used two-tailed Student’s *t* test, and a significant difference of *p* < 0.05 was verified. Statistical values were given as mean ± SEM.

## Results

### Free Ca^2+^ concentration in the solution for rapid cooling

We measured the contaminated free Ca^2+^ concentration in the solution used for RC containing 4.6 mM ATP and 20 μM fluo-3 with either fluo-3 or aequorin. The average free Ca^2+^ concentration was estimated to be 19 ± 2 nM (*n* = 11) measured with fluo-3 (protocol D1 in Fig. 3) and 20 ± 2 nM (*n* = 3) with aequorin at 3°C. There was no significant difference between the two values measured with fluo-3 and that with aequorin. Thus, the free Ca^2+^ concentration around SR was about 20 nM (pCa 7.7) before Ca^2+^ release was induced by RC.

### Fractional Ca^2+^ release induced by rapid cooling

**Fig. 4:**
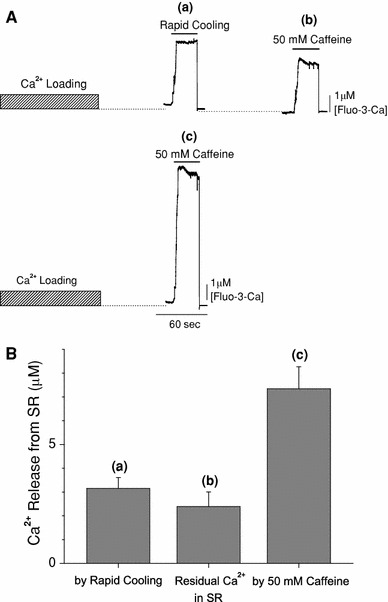
The fraction of SR Ca^2+^ released by rapid cooling. **A** Examples of fluorescence traces obtained with protocols C (*upper*) and B (*lower*). Fluo-3 fluorescence signals have been calibrated in terms of [Fluo-3-Ca]. With protocol C (*upper traces*), rapid cooling caused a rise of [Fluo-3-Ca] (*a*). After a brief wash with Ca^2+^-free solutions, subsequent application of 50 mM caffeine also caused an increase of [Fluo-3-Ca] (*b*) that reflected the release of residual Ca^2+^ in SR. With protocol B (*lower trace*), a large increase in [Fluo-3-Ca] was observed with the application of the solution containing 50 mM caffeine (*c*). At the end of assay periods in *a*–*c* (indicated by *horizontal bars*), rapid decreases of [Fluo-3-Ca] were due to closing an optical shutter, which terminated the fluorescence recordings. **B** Columns *a*–*c* summarise the Ca^2+^ release from SR (Δ[Fluo-3-Ca]) obtained from the type of experiment shown in *a*–*c*, respectively, in **A**. *Columns* show mean ± SEM of five preparations

Figure 4a shows typical traces of the fluorescence measurements with protocols C (upper) and B (lower) (Figs. 2, 3) obtained from the same preparation. After Ca^2+^ loading of the SR, RC induced a large increase in the [Fluo-3-Ca] in the capillary space (a). The preparation was briefly washed with Ca^2+^-free solutions (G10R and G1R, see protocol C in Fig. 3), and a subsequent application of 50 mM caffeine (plus 25 mM AMP) caused a substantial increase in [Fluo-3-Ca] (b), indicating that the SR still contained a significant amount of Ca^2+^ after RC. When the SR was reloaded with Ca^2+^ and the caffeine assay (50 mM caffeine plus 25 mM AMP) was repeated without RC in the same preparation, an increase in [Fluo-3-Ca] was greater by about a factor of two than that induced by RC (c).

From pooled data shown in Fig. 4b, the amount of SR Ca^2+^ released by RC (a) was, on average, 3.16 ± 0.46 μM. On the other hand, the residual SR Ca^2+^ after RC (b) was, on average, 2.39 ± 0.61 μM. This value was significantly smaller (*p* < 0.01) than the 4.19 ± 0.49 μM predicted by the total amount of releasable Ca^2+^ (*c* = 7.34 ± 0.93 μM) minus the amount Ca^2+^ released by RC (a). This difference is likely due to Ca^2+^ leakage from the SR into solutions that contained EGTA during 1.5 min washing periods inserted between RC and caffeine applications (see Fig. 3, protocol C). In saponin-treated cardiac muscles of mice, Morimoto et al. [13] reported a substantial Ca^2+^ leakage from the SR; about 50% of the SR Ca^2+^ content was lost when the preparation was perfused with the solution that contained 1 mM EGTA for 1.5 min at 22°C.

Thus, the fraction of Ca^2+^ released by RC was, on average, 44.7 ± 1.7% of the total SR Ca^2+^ content in 16 preparations.

### Rapid cooling with ATP opens the considerable SR Ca^2+^ release channels

**Fig. 5:**
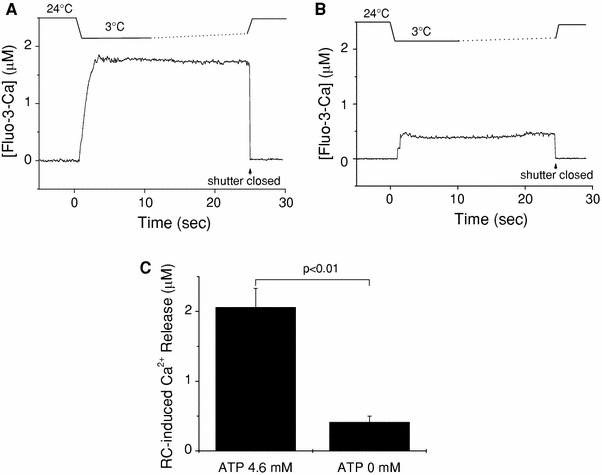
Rapid cooling-induced Ca^2+^ release in the presence or absence of ATP. **a**, **b** Traces show changes in solution temperature (*top*) and [Fluo-3-Ca] (*bottom*) in the presence (**a**) or absence (**b**) of ATP. The fluorescence recording was terminated by closing an optical shutter at the times indicated by *arrows*. **c** Summary of RC-induced Ca^2+^ release (Δ[Fluo-3-Ca]) estimated in the type of experiments shown in **a** and **b**. *Left* and *right columns* represent mean ± SEM of data obtained, respectively, in the presence (*n* = 16) and in the absence (*n* = 7) of ATP

With the standard solution containing 4.6 mM ATP (G0M1.5F, Table 1), a considerable amount of Ca^2+^ was released from the SR by RC (Fig. 5a), whereas in the absence of ATP (G0RM1.5F, Table 1) RC caused a much a smaller Δ[Fluo-3-Ca] signal (Fig. 5b), suggesting that Ca^2+^ release channels of SR hardly opened. The amount of Ca^2+^ released by RC in the presence and absence of ATP were 2.06 ± 0.27 and 0.41 ± 0.09 μM, respectively (Fig. 5c).

### Rapid cooling-induced Ca^2+^ release in the presence of ATP or AMP-PCP

**Fig. 6:**
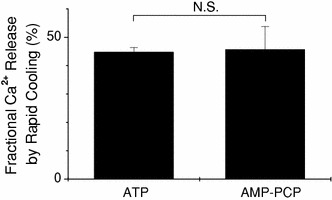
Fraction of SR Ca^2+^ released by rapid cooling in the presence of either ATP or AMP-PCP. Ca^2+^ release was induced by rapid cooling in the presence of 4.6 mM ATP (*left*, *n* = 16) or in the presence of 4.6 mM AMP-PCP (*right*, *n* = 4). The amount of Ca^2+^ released by rapid cooling was normalised to the total amount of releasable Ca^2+^ in the SR (*ordinate*). *Each bar* represents the mean ± SEM

The SR Ca^2+^ pump still works (albeit slowly) in the presence of ATP at 3°C [14]. Therefore, SR could reuptake a considerable part of the Ca^2+^ released by RC and might decrease the free Ca^2+^ concentration in the glass capillary lumen. Inhibition of the SR Ca^2+^ pump could lead to an increase in the free Ca^2+^ concentration around SR and might trigger the CICR. In order to examine a possible role of CICR in the RC-induced Ca^2+^ release, we compared the fraction of SR Ca^2+^ released by RC in the presence of ATP and AMP-PCP, a non-hydrolysable ATP analogue. The fraction of Ca^2+^ released by RC was 44.7 ± 1.7% with 4.6 mM ATP (see above) and was 45.6 ± 8.2% with 4.6 mM AMP-PCP (Fig. 6). There was no difference between these two values. The present results suggest that the RC-induced Ca^2+^ release is not influenced by hydrolysis of ATP, in other words, by the activity of SR Ca^2+^ pump.

### The Ca^2+^-induced Ca^2+^ release at 3°C

**Fig. 7:**
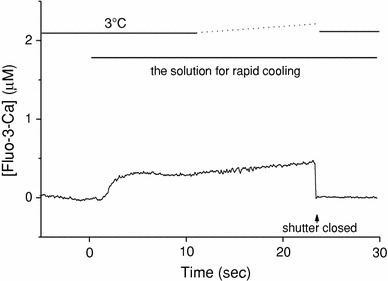
Ca^2+^ release from the SR induced by the solution for rapid cooling at 3°C. After Ca^2+^ loading of the SR, the temperature of the solution for the wash (G1R, Table 1) and the pre-assay solution (G0RM1.5F, Table 1) was gradually lowered from 24 to 3°C, and then the assay solution that contained ATP and fluo-3 (G0M1.5F) was applied at 3°C to the pre-cooled preparation as indicated at the top. Fluo-3 fluorescence signals were measured and were calibrated in terms of [Fluo-3-Ca]. An optical shutter was closed at a time indicated by an *arrow*

After Ca^2+^ loading in the SR, the temperature of the solution for the wash (G1R), which contains neither ATP nor Ca^2+^, was slowly lowered from 24 to 3°C for 1 min, and then the solution for RC (containing 4.6 mM ATP and ~20 nM free Ca^2+^) was applied at 3°C to the preparation (slow cooling). Then, we measured the change of Ca^2+^ concentration in a glass capillary. The typical data are shown in Fig. 7, and the mean value was 0.23 ± 0.10 μM (*n* = 5).

Because the temperature around the preparation was kept constant at approximately 3°C, the small Ca^2+^ release induced by the solution exchange was not due to cooling. Rather, it was thought that Ca^2+^ was released from the SR by changes in ATP and free Ca^2+^ concentrations (i.e., CICR). Aside from the initial rise, the [Fluo-3-Ca] signal also showed a gradual rise that started about 10 s after the solution exchange (Fig. 7). We might speculate that the CICR mechanism was gradually accelerated as the free Ca^2+^ concentration around the SR increased.

## Discussion

### Role of Ca^2+^ in the rapid cooling-induced Ca^2+^ release

In frog skeletal muscle, a slight increase in the intracellular Ca^2+^ concentration around the SR before RC was required for the RC-induced Ca^2+^ release. To increase the intracellular Ca^2+^ concentration before RC, a low concentration of caffeine was employed because the RC itself could not sufficiently increase the intracellular Ca^2+^ concentration for triggering a considerable Ca^2+^ release in skeletal muscles [4]. Therefore, the RC-induced Ca^2+^ release in skeletal muscles was assumed to be due to the enhancement of the CICR mechanism [5]. During RCC in frog skeletal muscle, the change in the aequorin light signal showing the intracellular Ca^2+^ concentration occurred in three phases. The first phase was a transient change of intracellular Ca^2+^ concentration accompanying slight tension. During the second phase, the light signal slowly increased as cooling produced maximum tension development. The third phase was an additional large light signal induced after the second phase, even though the tension was saturated. The second and third phases were more sensitive to low concentrations of procaine, an effective inhibitor for CICR, than the first phase [4].

On the other hand, pretreatment of the preparation with caffeine is not required for the initiation of RCC in mammalian cardiac and smooth muscles [2, 3]. In contrast to RCC in skeletal muscles, the amount of Ca^2+^ released by RC in ferret ventricular muscles was not influenced by the intracellular Ca^2+^ concentration before RC [6].

In the present study, in skinned cardiac muscles, the free Ca^2+^ concentration in the solution used for RC (G0M1.5F, see Table 1) was about 20 nM when estimated with either fluo-3 or aequorin, which seems lower than the level required to activate the CICR. It has been reported that the CICR is hardly activated at Ca^2+^ concentrations below 100 nM (i.e., pCa < 7) [15–17]. Thus, the contribution of CICR may be minor, if it exists at all, for the initiation of Ca^2+^ release induced by RC. This is supported by two lines of evidence. (1) When the preparation was perfused with the solution containing 20 nM free-Ca^2+^ and 4.6 mM ATP (G0M1.5F, Table 1) with the temperature set constant at 3°C (Fig. 6), the [Fluo-3-Ca] signal rose slowly with the delayed onset (~5 s after the solution exchange), and the [Fluo-3-Ca] signal was, on average, 0.23 ± 0.10 μM. This value was only about 10% of that achieved by RC (2.06 ± 0.27 μM). (2) The SR Ca^2+^ pump still works, albeit slowly, in the presence of ATP even at lower temperatures [14]. When the SR Ca^2+^ pump is completely inhibited (by replacement of ATP with AMP-PCP), the CICR may be enhanced because of the local increase in the free Ca^2+^ concentration around the SR. The fractional Ca^2+^ release induced by RC was unchanged even when ATP was replaced by AMP-PCP. Overall, the results suggest that CICR might not play an important role in the RC-induced Ca^2+^ release.

### Mechanisms of opening the Ca^2+^ release channels by rapid cooling

Global conformational changes upon binding ligands were observed with the gap junction protein [18] and with the nicotinic acetylcholine receptor [19]. Orlova et al. [20] demonstrated the three-dimensional structure of the rabbit skeletal muscle Ca^2+^ release channel in an open state using electron cryomicroscopy and angular reconstitution. In contrast to its closed state, in the open state reconstruction, a central cavity was revealed in the transmembrane region of the channel in the presence of Ca^2+^ and ryanodine. The opening of the channel is associated with a 4° rotation of its transmembrane region with respect to its cytoplasmic region and with significant mass translocations within the entire cytoplasmic region of the channel tetramer.

RC alone hardly opens the SR Ca^2+^ release channels. In the present study, RC could release considerable Ca^2+^ when ATP or AMP-PCP co-existed (Figs. 5, 6). Sitsapesan et al. [21] also confirmed that the SR Ca^2+^-release channels in the artificial lipid bilayer were not activated at low temperatures in the absence of cytosolic Ca^2+^ or a cardiotonic agent, sulmazole. Another study revealed that the presence of 10 μM cytosolic Ca^2+^ and 100 μM cytosolic ATP increased the mean open probability from 0.052 to 0.284 in sheep cardiac ryanodine receptor channels incorporated into planar phospholipid bilayers [22].

At low temperature and in the presence of Ca^2+^ [21], ryanodine, caffeine and adenine compounds [22], the Ca^2+^ release channels seem to be stable in an open state compared with that in closed state. In other words, low temperature is considered one of the so-called opening factors on the SR Ca^2+^ release channels. In cardiac, skeletal and smooth muscles, in the presence of one or more of those opening factors, the Ca^2+^ release channels might be stable in an open state. In addition, it was considered that the rapid lowering of the temperature may be important to synchronize the opening of a large population of Ca^2+^ release channels on SR membranes.

### Fractional amount of Ca^2+^ release by rapid cooling

Bers et al. measured intracellular Ca^2+^ transients during RCCs in guinea pig ventricular myocytes using the fluorescent Ca^2+^ indicator, Indo-1. They demonstrated that RC of myocytes from 22 to 0–1°C could release a large amount of Ca^2+^ ([Ca^2+^]_i_ > 10 μM) from the SR and suggested the use of RCCs as a useful means of assessing the SR Ca^2+^ content in intact cardiac muscle cells [23].

In our previous study, we estimated the fraction of SR Ca^2+^ released by the RC in intact papillary muscles of ferrets [6]. The intracellular free Ca^2+^ concentration was measured with aequorin, and Ca^2+^ release was first induced by RC (from 30 to 4°C) and subsequently by 15 mM caffeine. This experimental protocol was somewhat similar to that employed in the present study (Fig. 4a, upper). Both RC and subsequent application of caffeine caused transient rises in [Ca^2+^]_i_, and the peak changes in [Ca^2+^]_i_ induced by RC and caffeine averaged, respectively, 1.59 and 0.90 μM [6]. From these results, the fraction of SR Ca^2+^ released by RC was calculated to be 64% [=1.59/(1.59 + 0.9) × 100], which was higher than that estimated in the present study (44.7%; see “Results”). The difference may be attributed to the experimental methods employed in the previous study, in which peaks of Δ[Ca]_i_ measured with or without 15 mM caffeine were directly compared. The increase in apparent Ca^2+^ sensitivity was produced by caffeine in skinned cardiac and skeletal muscle fibres of the rat [24]. Furthermore, the complex of troponin (Tn) C with Tn I and Tn T resulted in an increase in the affinity of Tn C for Ca^2+^ in the presence of caffeine in the bovine heart [25]. Therefore, the amount of Ca bound to Tn C should be greater at any given [Ca^2+^]_i_. It follows that the use of Δ[Ca]_i_ measured during caffeine application would erroneously underestimate the total amount of Ca^2+^ released from the SR (Δ[Ca]_tot_), unless an appropriate correction was made. Consequently, a fraction of Δ[Ca]_tot_ induced by RC would have been overestimated in the previous study. In the present study, on the other hand, Δ[Ca]_tot_ was deduced by scaling Δ[Fluo-3 · Ca]_tot_ with factors separately estimated in the presence (2.78) and in the absence (1.80) of 50 mM caffeine (Fig. 1). Thus, we consider that the revised value in the present study (44.7%) is more relevant as an estimate of the fraction of SR Ca^2+^ released by RC.
